# The Inhibitory Effects of *Terminalia catappa* L. Extract on the Migration and Invasion of Human Glioblastoma Multiforme Cells

**DOI:** 10.3390/ph14111183

**Published:** 2021-11-19

**Authors:** Hsiao-Hang Chung, Ming-Ju Hsieh, Yih-Shou Hsieh, Pei-Ni Chen, Chung-Po Ko, Nuo-Yi Yu, Chiao-Wen Lin, Shun-Fa Yang

**Affiliations:** 1 Department of Horticulture, National Ilan University, Yilan 260, Taiwan; hhchung@niu.edu.tw; 2 Oral Cancer Research Center, Changhua Christian Hospital, Changhua 500, Taiwan; 170780@cch.org.tw; 3 College of Medicine, National Chung Hsing University, Taichung 402, Taiwan; 4 Graduate Institute of Biomedical Sciences, China Medical University, Taichung 404, Taiwan; 5 Department of Biochemistry, School of Medicine, Chung Shan Medical University, Taichung 402, Taiwan; csmcysh@csmu.edu.tw; 6 Institute of Medicine, Chung Shan Medical University, Taichung 402, Taiwan; peini@csmu.edu.tw (P.-N.C.); cbko1218@gmail.com (C.-P.K.); tonyturtle760310@hotmail.com (N.-Y.Y.); 7 Department of Neurosurgery, Tungs’ Taichung Metro Harbor Hospital, Taichung 433, Taiwan; 8 Department of Medical Research, Chung Shan Medical University Hospital, Taichung 402, Taiwan; 9 Institute of Oral Sciences, Chung Shan Medical University, Taichung 402, Taiwan; 10 Department of Dentistry, Chung Shan Medical University Hospital, Taichung 402, Taiwan

**Keywords:** glioblastoma multiforme, invasion, migration, brain cancer, *Terminalia catappa* L.

## Abstract

Glioblastoma multiforme (GBM) is one of the most aggressive and common types of brain tumor. Due to its high proliferation ability, a high lethality rate has been observed with this malignant glial tumor. *Terminalia catappa* L. (*T. catappa*) is currently known to have anti-inflammatory and anti-carcinogenesis effects. However, few studies have examined the mechanisms of the leaf extracts of *T. catappa* (TCE) on GBM cells. In the current study, we demonstrated that TCE can significantly inhibit the migration and invasion capabilities of GBM cell lines without showing biotoxic effects. Matrix metalloproteinases-2 (MMP-2) activity and protein expression were attenuated by reducing the p38 phosphorylation involved in the mitogen-activated protein kinase (MAPK) pathway. By treating with TCE and/or p38 inhibitor (SB203580), we confirmed that p38 MAPK is involved in the inhibition of cell migration. In conclusion, our results demonstrated that TCE inhibits human GBM cell migration and MMP-2 expression by regulating the p38 pathway. These results reveal that TCE contains potent therapeutic compounds which could be applied for treating GBM brain tumors.

## 1. Introduction

Brain tumors are divided into two major types: cancerous and benign tumors. In the cancerous type, the glioblastoma multiforme (GBM) is the most common type of brain tumor in humans [1]. Due to its high proliferation rate and invasiveness, GBM is recognized as a lethal brain tumor that shows a 2-year survival rate of less than 5% [2,3]. The standard treatments for GBM are surgery, radiotherapy and chemotherapy. Combinations of radiotherapy and the conventional chemotherapy drug, temozolmide (TMZ), show a higher survival rate than radiotherapy alone [4,5]. Currently, multimodal treatment including feasible surgical resection, radiotherapy and adjuvant TMZ is still not sufficient, exhibiting survival rates of only around 14.6 months after diagnosis [6]. The chemotherapy drug TMZ can cause significant toxic side effects in nearly 20% of treated patients [7]. Due to this limitation of TMZ, the development of a new approach for GBM treatment is an important task today.

The high lethality of GBM is largely caused by its infiltrative invasion and recurrence at adjacent or distant regions of the brain after surgery [8,9]. The metastasis of cancer cells commences with the invasion of adjacent cells and the formation of new tumors [10,11,12]. The hydrolysis of the extracellular matrix (ECM) is a critical process in cell metastasis that promotes the mobility and metastasis of cancer cells [13,14,15]. Various secreting proteases are involved in this hydrolysis process; one of the major protease groups is referred to as the matrix metalloproteinases (MMPs) [16,17,18,19]. MMP-2 (gelatinase A) is a type IV collagenase that plays a vital role in glioma carcinogenesis [20,21]. Clinical studies have indicated that high levels of MMP-2 activity are associated with intracerebral invasion and metastasis of GBMs [20]. Numerous in vitro studies have revealed that suppression of MMP-2 expression contributes to the inhibition of cell migration in GBM cells [22,23,24,25]. Thus, inhibition of MMPs could be effective for preventing the metastasis of cancer cells.

Currently, enormous numbers of studies in different fields, such as pharmacology, medical science, food science, agriculture, etc., have focused on the application of plant secondary metabolites. Pharmacological effects in the prevention or intervention of disorders is a most attractive topic these days. *Terminalia catappa* L. (*T. catappa*) is a large tree species belonging to the family *Combretaceae* and is widely distributed over the tropical and subtropical regions of Asia and Australia. It grows quickly and propagates easily in suitable environment via seeds. Various parts of this tree, most commonly leaves, barks and fruits, have been applied as traditional remedies, and are reputed for treating dermatitis and fever in India, Malaysia and the Philippines [26]. More recently, a number of plant compounds including flavonoids, triterpenoids and hydrolysable tannins have been identified from the chloroform extract or water extract of *T. catappa* leaves [27,28,29,30]. These results might also explain the anti-oxidative, anti-inflammatory, anti-carcinogenesis and hepatoprotective effect of water extracts of *T. catappa* leaves that have been found in many research reports [31,32,33]. Therefore, further studies on the leaf extracts of *T. catappa* (TCE) and their applications would benefit human health. Previous studies from our research team have demonstrated that TCE can inhibit the metastasis of Lewis lung carcinoma, oral squamous cell carcinoma and hepatocellular carcinoma in vitro and/or in vivo [31,34]. In this study, we characterized the anti-carcinogenesis effect of TCE on malignant primary brain tumor GBMs and tried to understand its inhibitory mechanisms. The results showed that TCE can also inhibit the migration and invasion of GBM by suppressing MMP-2 expression.

## 2. Results

### 2.1. Effects of TCE on Cell Viability in U251 and GBM8401 Cell Lines

**Figure 1:**
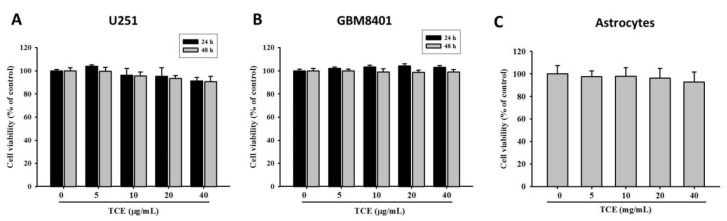
Effects of *T. catappa* L. extract (TCE) on cell viability. U251 (**A**) and GBM8401 (**B**) cells were treated with different concentrations (0, 5, 10, 20 and 40 μg/mL) of TCE for 24 and 48 h before being subjected to an MTT assay for cell viability. (**C**) Immortalized human astrocyte cells were treated with TCE for 24 h before being subjected to an MTT assay for cell viability. The values shown represent the means ± SD of at least three independent experiments.

By using the MTT assay, the cytotoxic effects of TCE at various concentrations (0–40 μg/mL) following its application for 24 and 48 h on U251 and GBM8401 cells were characterized. The results clearly showed that U251 and GBM8401 cell lines were not significantly affected at all treatment concentrations after 24 h and 48 h of treatment (Figure 1A,B). Moreover, TCE treatment did not reduce cell viability in immortalized human astrocyte cells (Figure 1C). Therefore, the tested range of TCE concentrations did not exhibit any toxic effects and so was applied for the following experiments.

### 2.2. Effects of TCE on Migration and Invasion of U251 and GBM8401 Cell Lines

**Figure 2:**
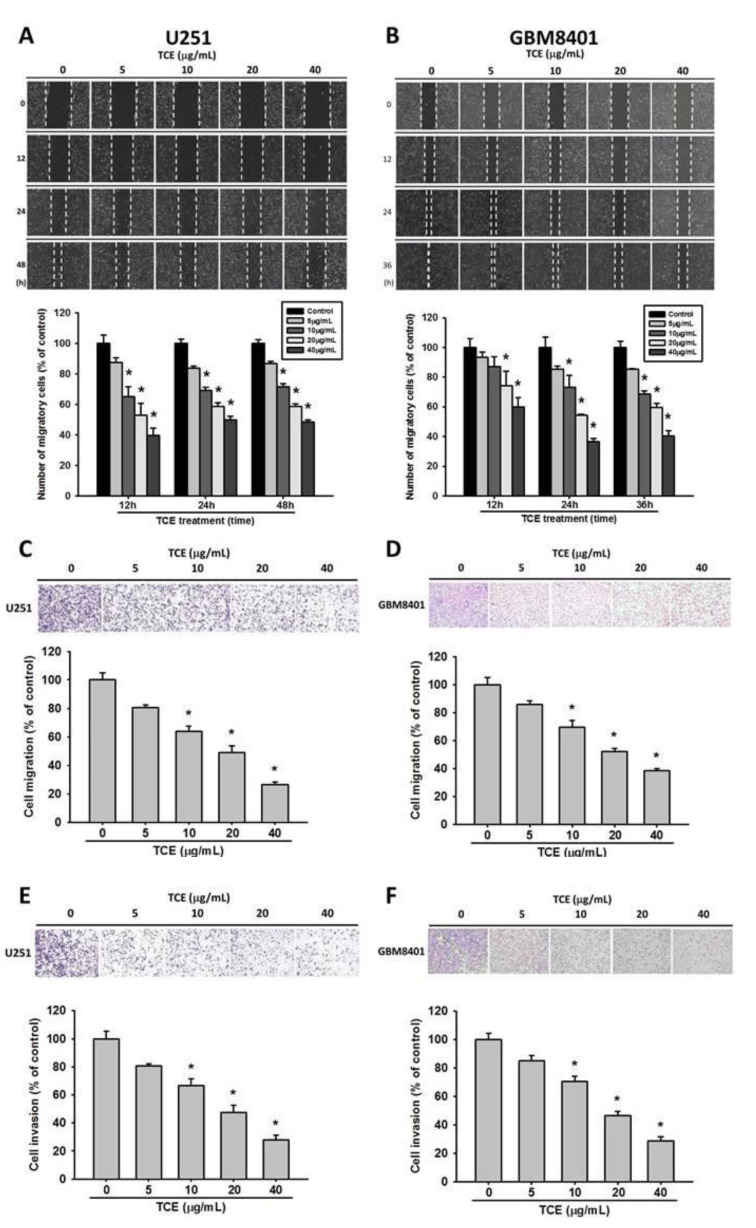
Effects of *T. catappa* L. extract (TCE) on migration and invasion of U251 and GBM8401 cells. The motility of U251 (**A**) and GBM8401 (**B**) cells were assessed by in vitro wound closure assay with different concentrations of TCE (0, 5, 10, 20 and 40 μg/mL) at 12, 24 and 48 h. Cell migration (**C**,**D**) and invasion (**E**,**F**) were measured using a Boyden chamber for 24 h with polycarbonate filters. * *p* < 0.05, compared with the vehicle group.

To investigate the motility of U251 and GBM8401 cell lines after TCE treatment, a scratch-wound assay was performed. A significant inhibition at 12 h was found with the higher dosages of TCE, at 20 and 40 μg/mL (Figure 2A,B), and all TCE-treated samples also exhibited significant inhibition (*p* < 0.05) after 48 h. Both cell lines demonstrated dose and time-dependent effects on their motility. Moreover, using a Boyden chamber-based assay, we found that the migration and invasion ability of both glioma cell lines were decreased significantly by treatment with TCE (*p* < 0.05), except at the lowest concentration (5 μg/mL; Figure 2C–F). These outcomes revealed that TCE is able to prohibit migration and invasion in both tested glioma cell lines.

### 2.3. TCE Inhibits the Enzyme Activity and Protein Expression of MMP-2

**Figure 3:**
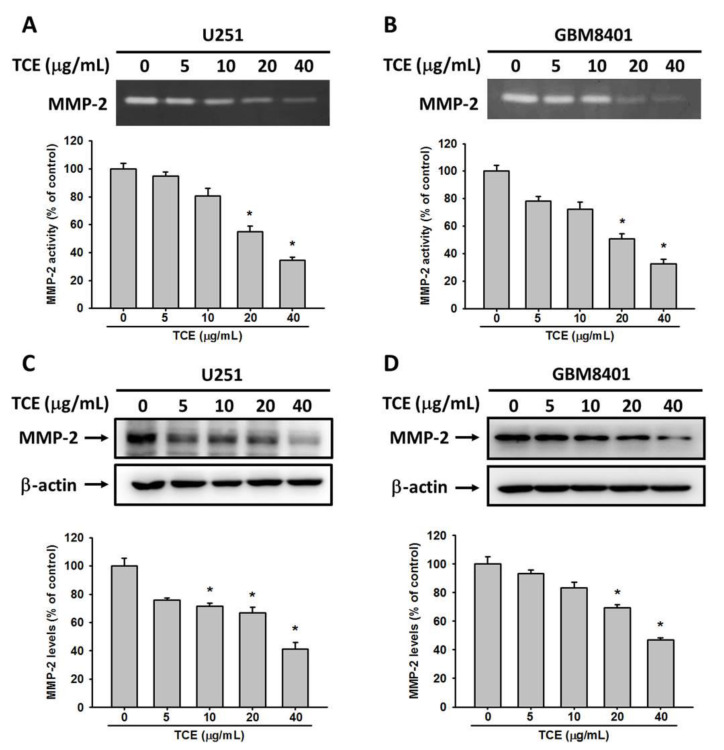
Effects of *T. catappa* L. extract (TCE) on the enzymatic activity and protein expression of MMP-2. A gelatin zymography assay was applied for the measurement of MMP-2 activity in TCE-treated (**A**) U251 and (**B**) GBM8401 cells (0, 5, 10, 20 and 40 μg/mL). The expressions of MMP-2 protein during the same treatments of (**C**) U251 and (**D**) GBM8401 cells were assessed by western blot. The values represent the means ± SD of at least three independent experiments. * *p* < 0.05, compared with the vehicle group.

MMPs are the major proteases involved in the cancer cell migration. Thus, we would like to clarify the role of MMP-2 in the TCE inhibition of glioma cells. The enzyme activity and protein expression of MMP-2 were characterized by a gelatin zymography assay and Western blotting, respectively. MMP-2 activity gradually decreased along with the increase of TCE concentrations in both cell lines (Figure 3A,B). TCE reduced the MMP-2 enzymatic activity by 66% and 59% in U251 and GBM8401 cells, respectively, at the highest concentration (40 μg/mL). MMP-2 protein expression was downregulated in both TCE-treated cells, which corresponds with the enzymatic activity assay (Figure 3C,D). Thus, inhibitory effects on migration in TCE-treated U251 and GBM8401 cells were found to occur via the suppression of MMP-2 activity and protein expression.

### 2.4. Effects of TCE on Signaling Cascades in U251 and GBM8401 Cell Lines

**Figure 4:**
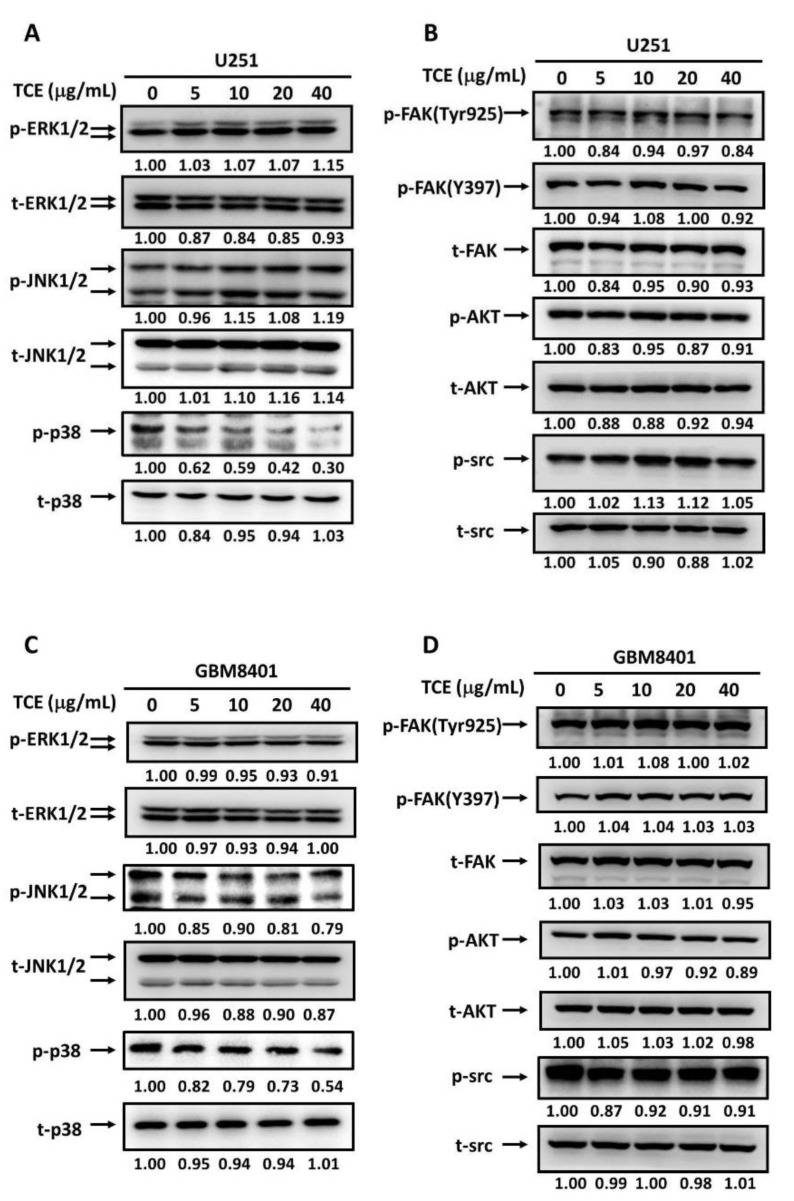
Effects of *T. catappa L*. extract (TCE) on signaling cascades in U251 and GBM8401 cells. After 6 h culture with various concentrations of TCE (0, 5, 10, 20, and 40 μg/mL), the lysates of U251 and GBM8401 cells were subjected to SDS-PAGE followed by a Western blots assay with (**A**,**C**) anti-ERK1/2, anti-JNK1/2, anti-p38 and (**B**,**D**) anti-FAK, anti-AKT, anti-Src (total and phosphorylated) antibodies.

Based on the results above, we wanted to investigate the regulatory mechanisms of TCE-treated cell signal transduction. Given that the MAPK pathway is involved in the regulation of MMP-2 expression, we therefore examined the levels of phosphorylated ERK1/2, JNK1/2, p38 in U251 and GBM8401 cells after TCE treatment. Results showed a significant decrease in p38 phosphorylation, while neither phosphorylation of ERK1/2 nor JNK1/2 activation was observed (Figure 4A,C). In addition, the FAK/Src and AKT pathways have previously been associated with increased tumor metastasis and upstream signaling pathways of MMP-2 expression. However, the phosphorylation levels of FAK/src and AKT expression in U251 and GBM8401 cells with or without the addition of TCE were nearly unchanged (Figure 4B,D). Therefore, we suggested that the suppression of MMP-2 by TCE was regulated via a component of the mitogen-activated protein kinase (MAPK) pathway—p38.

### 2.5. Effects of TCE and p38 Inhibition in U251 Cell Lines

**Figure 5:**
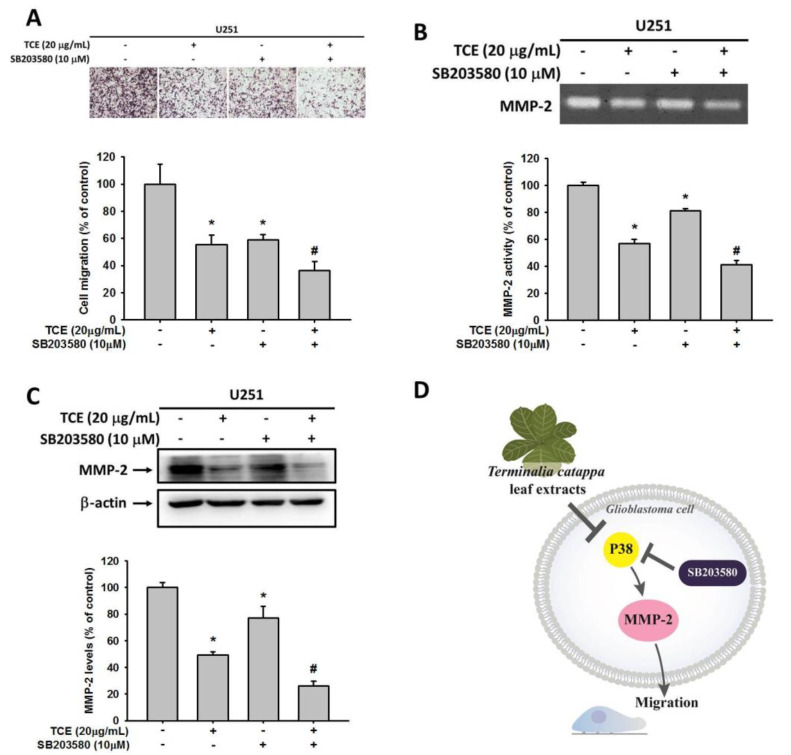
Effects of *T. catappa* L. extract (TCE) and p38 inhibitor (SB203580) on MMP-2 activity, protein expression and migration of U251 cells. U251 cells were treated with SB203580 combined with or without 20 μg/mL of TCE for 24 h. Analysis of (**A**) the migration of U251 cells was assessed as described in Section 4. A gelatin zymography assay was used for measurement of MMP-2 activity (**B**) and protein expression (**C**). (**D**) Schematic illustrates the mechanisms by which TCE inhibits GBM cell migration through the p38 pathway. * *p* < 0.05, compared with the vehicle group. # *p* < 0.05, compared with the TCE-treated group.

To further confirm the role of p38 in the TCE-induced anti-migration of glioma cells, the p38 inhibitor SB203580 was utilized and combined with TCE at 20 μg/mL for the following assays. A Boyden chamber-based assay showed that application of either the inhibitor or TCE alone could decrease U251 cells’ migrative ability. Combined TCE and SB203580 significantly reduced this migrative ability more than by treatment with either alone (Figure 5A). Similar results were observed on MMP-2 activity and protein expression levels, which were dramatically reduced with the combination treatment of inhibitor and TCE, as shown by zymography and a Western blot assay (Figure 5B,C). Sequential assays with the SB203580 inhibitor clearly indicated that p38 is involved in TCE-induced anti-migration in the glioma U251 cell line (Figure 5D).

## 3. Discussion

According to the 2021 World Health Organization (WHO) classification, GBM has been recognized as one of the most aggressive type of primary brain cancer [35]. Only a 9.8% 5-year survival rate of patients is found with adjuvant chemotherapy of TMZ following safe surgical resection [36]. The high proliferation of gliomas is the main factor causing the death of patients. More and more studies have demonstrated that natural compounds have the potential to inhibit the proliferation, migration and/or invasion of various tumor cell lines as novel anti-cancer drugs [37]. Therefore, preventing metastasis is one critical approach for cancer disease treatment. In the current study, the results exhibited that treatment with TCE suppressed the migration and invasion of U251 and GBM8401 cell lines (Figure 2), and showed no cytotoxicity at the applied concentrations (Figure 1). Through inhibition of the phosphorylation of p38 expression in the MAPK pathway, TCE lowered the expression of MMP-2 protein and its enzymatic activity. We deemed that TCE could inhibit cell migration by interfering with MMP-2 expression by inhibiting the activation of p38 phosphorylation.

*T. catappa* plants have long been used in folk medicine and also show anti-cancer capabilities in many in vitro and in vivo studies [31,34,38,39,40]. In a previous study, two triterpenoids, ursolic acid and asiatic acid, were identified from TCE [41]. In a recent study, Conway et al. provided evidence that ursolic acid has a high capacity for inducing GBM cell death and inhibiting GBM cell migration [42]. Moreover, ursolic acid was shown to increase sensitivity to TMZ in TMZ-resistant GBM cells [43]. Based these findings, ursolic acid, one of the most abundant components of TCE, could be applied to act as an inhibitor of GBM cell migration. However, further investigations are required to fully elucidate whether TCE can pass through the blood–brain barrier.

The degradation of the ECM is considered an important marker of the progress of metastasis [13,14,44,45,46]. Numerous previous studies have revealed that this process is catalyzed by proteins secreted by cancer cells and allows cancer cells to migrate to other tissues or organs [17]. It has been demonstrated that extracellular matrix (ECM) remodeling regulates GBM cells’ migratory and infiltrative potential [47]. The MMPs are the major secreted enzymes that are involved in different types of malignant tumors [48]. MMP-2, also known as gelatinase A, is known to be expressed in most tissues and cells [49]. It has also been shown to play a key role in the invasion and metastasis of different types of human cancer cells [50,51,52,53,54]. A few studies have found high expression of MMP-2 has in high-grade astrocytic tumors in comparison to normal brain tissue [55,56]. In addition, the expression levels of MMP-2 were significantly elevated in TMZ-resistant GBM cells in comparison to parental GBM cells [57]. The use of novel compounds derived from natural products is suggested as a potential solution for the treatment of GBM [58]. Jiang et al. revealed that tetrandrine, which is isolated from the root of *Stephania tetrandra* S. Moore, inhibits GBM8401 cancer cell migration and invasion in vitro [59]. A novel caffeic acid amide derivative, PT93, suppresses MMP-2 and MMP-9 expression in human GBM cell lines [55]. Our results reveal that TCE inhibits the migration abilities of GBM cells by suppressing MMP-2 expression. Thus, TCE may serve as a potential agent for GBM therapy.

The MAPK network has been shown to be activated in over 88% of gliomas, regulating gliomagenesis and progression [60,61]. Research has shown that targeting MAPK-interacting kinases (MNK1/2) downstream of the MAPK-signalling pathway may be an effective strategy for treating GBM [62]. Moreover, Wu et al. reported that knockdown uncoupling protein 2 reduces glioblastoma cell invasiveness by inhibiting the p38 pathway [63]. Similarly, our study reported p38 MAPK activity as affecting human glioma cell migrative ability via TCE, which controls the metastatic processes of glioma cells. However, our studies only focused on the mechanistic roles of human U251 and GBM8401 glioma cell lines, and the effect of TCE on GBMs was not studied in vivo. Further in vivo investigations are needed to verify the anti-metastatic effects of TCE in GBM in the future.

## 4. Materials and Methods

### 4.1. Preparation of T. catappa L. Extract (TCE)

*T. catappa* L. leaves were purchased from local herb stores in Taichung, Taiwan. The plant material was identified at the Department of Biochemistry, Chung Shan Medical University in Taichung by Dr. Yih-Shou Hsieh. Ethanol was utilized for the preparation of *T. catappa* L extracts (TCEs) as described previously [34]. In brief, 100 g of the air-dried leaves were extracted twice by 50% ethanol (500 mL) in a flask which was boiled at 70 °C for 24 h. After filtration of the pooled extract, the solvent was removed using a rotary evaporator under a low pressure. The condensed extract was then lyophilized and stored at −20 °C. The chemical profile of the TCE was also characterized using HPLC–MS as described previously [34]. Analysis of TCE using the HPLC–MS system revealed one major peak with a retention time of 11.13 min. The main product peak was then subjected to mass spectrometry and ellagic acid (the major peak was at the *m/z* 301.03); hydrolysable tannins (punicalin and punicaligin) and gallic acid were identified, which were consistent with previous reports [64,65,66]. The results revealed that ellagic acid and hydrolysable tannins are the major chemical constituents of TCE. In this study, the TCE extract was prepared with 50% ethanol under the same conditions and the chemical profile was similar to our previous study [34].

### 4.2. Cell Lines and TCE Treatment

Two human glioma cell lines, U251 and GBM8401, utilized for the TCE inhibitory experiments were purchased from the BCRC Center, Inc., Hsinchu, Taiwan. Both cell lines were cultured in RPMI-1640 medium supplemented with 10% fetal bovine serum, 2 mM L-glutamine, 100 μg/mL streptomycin and 100 U/mL penicillin at 37 °C in a humidified 5% CO_2_ incubator. Immortalized human astrocyte cells were purchased from Creative Biolabs, Inc., Shirley, NY, USA. The TCE powder was dissolved in 50% DMSO to prepare a serial stock of 0, 5, 10, 20 and 40 μg/mL for treatments. The p38 inhibitors (SB203580; 10 μM) were also utilized to reveal if the inhibitory effects of TCE were produced via the MAPK pathway.

### 4.3. Cell Viability Assay

For evaluating the cytotoxicity of TCE, we applied a colorimetric assay using a tetrazolium dye, 3-(4,5-dimethylthiazol-2-yl)-2,5-diphenyltetrazolium bromide (MTT), to determine cell viability. Both U251 and GBM8401 cells were seeded in 24-well plates at a density of 6 × 10^4^ cells/well and treated with TCE at 0, 5, 10, 20 and 40 μg/mL for 24 h under the same culture conditions. After the TCE treatments, the medium was removed and washed by PBS. The attached cells were further incubated with 20 μL of 5 μg/mL MTT (Sigma chemical Co., St. Louis, MO, USA) at 37 °C for 4 h. The number of viable cells was evaluated by the production of formazan, which was measured spectrophotometrically at 563 nm.

### 4.4. In Vitro Wound Closure

U251 and GBM8401 cells (2 × 10^5^ cells/well) were plated in 6-well plates for 24 h and wounds were produced by scratching with a pipette tip. The wounded cells were then incubated in RPMI medium and treated with TCE (0, 5, 10, 20 and 40 μg/mL). Images were recorded at 0 (immediately after wounding), 12, 24 and 48 h after treatment using a phase-contrast microscope (×100).

### 4.5. Migration and Invasion Assays

The assays for characterization of cell migration and invasion were as described in Chu et al. [34]. After treatment with or without TCE (0, 5, 10, 20 and 40 μg/mL) or in combination with the p38 inhibitor SB203580 (10 μM) for 24 h, surviving cells were collected and seeded onto the upper Boyden chamber (Neuro Probe, Cabin John, MD, USA) at 10^4^ cells/well in serum-free medium.

### 4.6. Assessment of MMP-2 by Gelatin Zymography

The activity of MMP-2 in the medium was measured by gelatin zymography assays, as described previously [40]. The cultured medium was collected after TCE treatment (0, 5, 10, 20 and 40 μg/mL) or in combination with the p38 inhibitor SB203580 (10 μM), and subjected to 0.1% gelatin-8% SDS-PAGE electrophoresis.

### 4.7. Western Blot Analysis

Total cell lysates from different treated cells were prepared. Equal amounts of protein samples (20 μg) from each cell lysates were separated by SDS-PAGE on 10% polyacrylamide gels and electrotransferred onto polyvinylidene fluoride (PVDF) membranes (Millipore, Belford, MA, USA). After blocking, the PVDF membranes were incubated with primary antibodies as described previously [34].

### 4.8. Statistical Analysis

The SigmaStat 2.0 software package (Jandel Scientific, San Rafael, CA, USA) was employed for statistical analyses. Differences between untreated and TCE-treated groups were calculated by Student’s *t*-test, and a *p* value of < 0.05 was considered statistically significant.

## 5. Conclusions

In conclusion, the present study demonstrated that TCE at noncytotoxic concentrations (0–40 μg/mL) noticeably reduced the cell migration and invasion of human GBM cells (U251 and GBM8401 cell lines). Moreover, TCE inhibits cell migration and MMP-2 expression by regulating the p38 pathway. Thus, our results have put forward evidence for TCE being a potential agent for clinical application in the inhibition of the migration and invasion of human glioma cells.

## Data Availability

Data is contained within the article.
